# Quantifying Weight Loss Prior to Pancreatic Cancer Diagnosis: A Systematic Review and Meta‐Analysis

**DOI:** 10.1002/cam4.71997

**Published:** 2026-05-31

**Authors:** Claire A. Price, Debbie Cooke, Freda Mold, Nadia A. S. Smith, Martyn Winn, Agnieszka Lemanska

**Affiliations:** ^1^ University of Surrey, School of Health Sciences Surrey UK; ^2^ National Physical Laboratory Middlesex UK; ^3^ Royal Surrey NHS Foundation Trust, Scientific Computing Guildford UK; ^4^ TÜV SÜD UK, Chadwick House Warrington UK; ^5^ Science and Technology Facilities Council, Scientific Computing Department, Research Complex at Harwell Didcot UK; ^6^ Imperial Clinical Trials Unit, Imperial College London London UK

## Abstract

**Introduction:**

Pancreatic cancer has a high mortality rate due to late diagnosis and presentation with non‐specific symptoms like weight loss. Quantifying weight loss more precisely could enhance its utility as a diagnostic marker.

**Methods:**

In March 2025, we systematically searched PubMed, Embase, Web of Science, The Cochrane Library, and Scopus for studies reporting quantitative pre‐diagnosis weight loss in pancreatic cancer, with no date restrictions. The protocol was registered with PROSPERO (CRD42022302985). Primary outcomes were weight loss (kg) and BMI change (kg/m^2^). Meta‐analyses provided pooled estimates with 95% confidence intervals, displayed in forest plots. We conducted stratified analyses at the study level by diabetes status, cancer type, self‐reported weight change, and timing of weight loss. Subgroup analyses considered publication year, country, study design, mean age, and percentage of male participants. Study quality and bias were assessed using the ROBINS‐I framework.

**Results:**

The analysis included 25 studies (25,971 participants). In random‐effects meta‐analysis, we found that on average participants lost 5.9 kg [95% CI 4.7 to 7.1] of body weight. The average BMI change was −2.5 kg/m^2^ [95% CI −3.0 to −2.1]. There was significant heterogeneity between studies for both weight loss (I^2^ = 99.2%) and BMI change (I^2^ = 82.6%). For weight loss in kg, heterogeneity was partially explained (*p* < 0.001) by diabetes status (0.15%), weight loss timing (15.5%), whether weight loss was self‐reported (11.4%) and publication year (13.1%). For BMI change heterogeneity was partially explained (*p* < 0.001) by pancreatic cancer type (1.3%), whether BMI change was self‐reported (49.6%) and publication year (49.5%). Post hoc pairwise comparisons of moderators for stratified analysis were not significant (*p* < 0.0125). The certainty of evidence was moderate due to high heterogeneity between studies.

**Conclusions:**

This meta‐analysis provides a reference for expected weight loss in pancreatic cancer, aiding clinical practice and improving early detection models.

## 1 Introduction

Although relatively rare, pancreatic cancer has a high mortality rate, with a 5‐year survival of just 3%–15% [1, 2, 3, 4]. This means it is the fifth leading cause of cancer‐related deaths in the UK [5], and its proportion of cancer mortality is expected to rise [6, 7, 8, 9], potentially exceeding breast cancer deaths in Europe by the 2030s [10, 11]. This high mortality is largely due to late‐stage diagnosis in 80% of cases, when the cancer has already spread and is no longer curable [12, 13, 14, 15, 16, 17]. As a result, early detection and timely diagnosis are critical to improving patient outcomes [18].

However, there are currently no reliable population‐based screening tests for pancreatic cancer [19, 20, 21, 22], and pancreatic cancer is often called a “silent killer” as it is difficult to detect. Diagnosis often relies on presentation of non‐specific symptoms to a GP. These symptoms include weight loss, which is reported by 70%–75% of patients before diagnosis [23, 24, 25, 26, 27, 28]. As a result, weight loss is a key diagnostic marker and has been incorporated into several data‐driven algorithms for early detection [29, 30, 31, 32, 33]. However, definitions vary: some models use arbitrary cut‐offs (e.g., 5% body weight), while others treat it as a continuous variable. A clearer, standardised understanding of weight loss is essential to refine and calibrate these predictive models for better accuracy and clinical application.

Weight loss in pancreatic cancer is often severe, typically happening one to two years before diagnosis [12, 23, 24, 25, 26, 32], with body composition changes beginning up to five years earlier [24, 27, 34]. Although this has been widely reported, the evidence has not yet been systematically synthesised. Unintentional and unexplained weight loss already forms a part of the strategy for referrals for suspected pancreatic cancer in the UK for people with new‐onset diabetes [31]. As weight loss often appears earlier than other disease‐specific symptoms such as jaundice [24, 25], understanding the pattern and extent of weight loss is crucial to improve its utility as a diagnostic marker. While weight loss is a non‐specific symptom common to many cancers, this study focuses on quantifying it specifically in pancreatic cancer to provide clinicians with a reference point for early detection. The aim of this review was to quantify pre‐diagnostic weight loss in pancreatic cancer. The objectives were to undertake a systematic review of epidemiological studies reporting weight changes before diagnosis, followed by a meta‐analysis to quantify the weight loss.

## 2 Methods

### 2.1 Protocol

The protocol for this systematic review was prospectively registered at the International Prospective Register of Systematic Reviews, PROSPERO (Registration ID No. CRD42022302985) [35]. Minor deviations from the protocol are listed in Supporting Information 1. The Preferred Reporting Items for Systematic Reviews and Meta‐Analyses (PRISMA) guidelines [36, 37] were followed to assist in structuring this review [Supporting Information 2 and 3].

### 2.2 Search Strategy

A systematic literature search was conducted in March 2025. Five databases were searched: PubMed, EMBASE, Scopus, Web of Science and The Cochrane Library, without imposing any restrictions on publication date. A reproducible search strategy was created which utilised index/MeSH (Medical Subject Headings) and a string of keyword terms (“Pancreas OR Pancreatic”) AND (“Ductal Adenocarcinoma OR PDAC OR Cancer OR Malignancy OR Adenocarcinoma OR Tumour OR Tumor”) AND (“Weight OR BMI OR Body Mass Index”) AND (“loss OR decrease OR change”) AND (“diagnos*”). The full search strategy can be found in Supporting Information 4.

There were no restrictions or filters applied at the electronic database search stage. In addition to the electronic database search, additional articles were identified by reviewing the references of eligible articles. We also applied the “similar articles” function in PubMed.

### 2.3 Inclusion and Exclusion Criteria

#### 2.3.1 Inclusion Criteria Based on PICOS

The inclusion criteria were constructed based on the population, intervention, comparator, outcome and study design (PICOS) framework [36, 37, 38], as outlined by the Cochrane Handbook [39].

(1) Population: Adults with pancreatic cancer diagnosis; (2) Comparison: None; (3) Outcome: Quantitative data on weight loss before pancreatic cancer diagnosis reported as (a) an amount in units (e.g., kg, lb), (b) BMI change, or (c) percentage loss from baseline; (4) Study design: Case–control, nested case–control, cohort, and any observational or retrospective studies of medical records.

#### 2.3.2 Exclusion Criteria

(1) Reports of weight loss without an appropriate measure of dispersion (standard deviation (SD), standard error (SE), confidence interval (CI), interquartile range (IQR) or range); (2) Weight loss during pancreatic cancer treatment or after diagnosis; (3) Benign pancreatic tumours; (4) Studies with fewer than 10 pancreatic cancer patients (due to possible small study effects); (5) Review articles, meta‐analysis, letters, comments, editorials, case reports, abstracts, or conference proceedings; (6) Animal studies; (7) Duplicated papers; (8) Non‐English full text; and (9) Non‐accessible full text.

### 2.4 Study Selection

Using the above search strategy, 6754 articles were retrieved. Duplicate articles were removed, and the titles and abstracts were screened for eligibility by CP and AL. After excluding ineligible articles based on the title and abstract, and adding studies identified by searching the references of the eligible articles, 328 articles were retrieved. The full text of these records was examined by CP, with AL independently reviewing 10% (selected at random). Inclusion queries were resolved through discussion at team meetings, and all team members agreed on the final selection.

**FIGURE 1:**
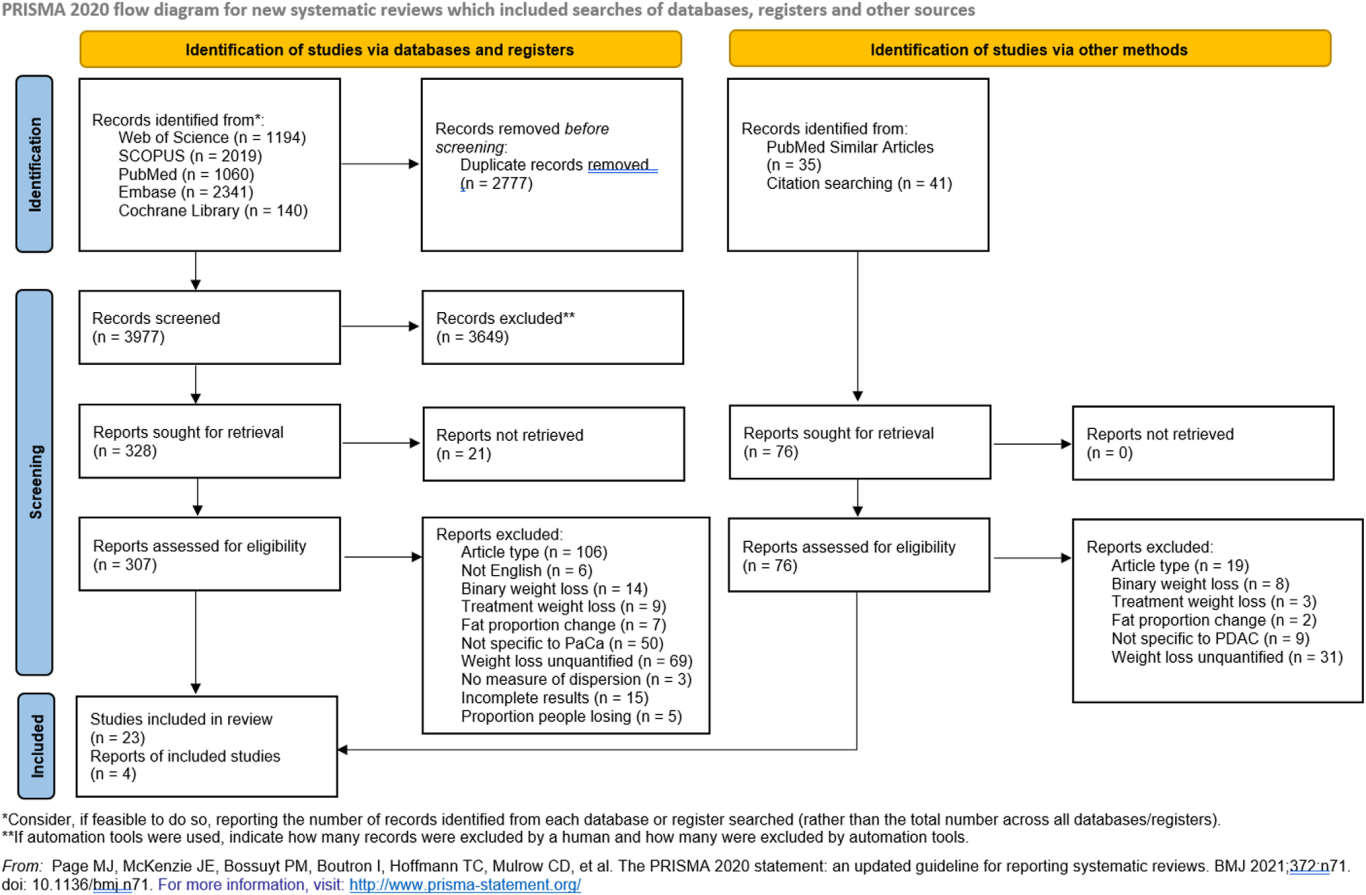
PRISMA flowchart of describing included/excluded articles. PaCa, Pancreatic Cancer; PDAC, Pancreatic Ductal Adenocarcinoma.

During this stage of screening, we contacted the authors of 15 studies where the results reported in the original papers were incomplete (e.g., only presenting the results graphically, not providing a baseline weight to allow conversion of percentage loss into loss in kg). However, the authors were unable to provide the complete results, so these studies were also excluded from the meta‐analysis. At the end of this screening, 27 papers remained. The number of articles included at each stage of screening is summarised in Figure 1, PRISMA flowchart.

### 2.5 Risk of Bias

Studies were evaluated using Risk Of Bias In Non‐randomised Studies of Interventions (ROBINS‐I) [40]. The tool includes 7 domains of bias due to (1) confounding, (2) participant selection, (3) classification of exposure/intervention, (4) misclassification/deviation of exposure/intervention during follow‐up, (5) missing data, (6) measurement outcomes, and (7) selective reporting of results. The overall risk‐of‐bias judgement for each study was summarised as “low risk” of bias if the study received the assessment of “low risk” for all domains, and “high risk” of bias if the study was judged to be at “high risk” in at least one domain. Studies with insufficient information to assess the risk of bias for some domains were categorised into “some concerns”. We excluded two studies due to concerns about bias, leaving a total of 25 eligible for inclusion in the meta‐analysis. Details of this evaluation for each study are shown in Supporting Information 5.

### 2.6 Data Extraction

Data extraction was carried out using an extraction spreadsheet, consisting of the following variables: Title, authors, year of publication, Digital Object Identifier (DOI), country, source (e.g., database, journal), study design, methodology, and sample size. The following variables related to weight loss were also extracted (all with associated measures of dispersion): Weight loss before diagnosis, BMI change before diagnosis, percentage weight change, baseline and diagnosis weights, and baseline and diagnosis BMI. In addition, the following variables were extracted if reported: Whether the reported weight loss was from medical records or self‐reported, diabetes status (including definition and duration), stage of cancer, location of tumour (pancreas head, body or tail), pancreatic cancer type (Pancreatic Ductal Adenocarcinoma (PDAC) or all pancreatic malignancies), weight loss time period and any other key messages not already recorded.

Many studies focused on the most prevalent type of pancreatic cancer, PDAC, whereas others included all pancreatic malignancies. This was recorded as pancreatic cancer type during data extraction. Late‐stage disease was classified as stage IIB+. Diabetes status refers to whether someone has diabetes or not. Some studies do not differentiate by diabetes type; therefore, analysis was restricted to the presence/absence of diabetes and not stratified by type. Some studies reported changes in weight split by diabetes status. In these cases, the statistics for the whole cohort were used. If weight loss even partly relies upon self‐reported measurements of previous or usual weight, then this is recorded as self‐reported weight loss. When data for multiple weight loss periods were available, 12 months was chosen as this is the most frequently reported weight loss period, so studies can be most easily combined/compared. Several studies only reported “usual adult weight” and not a specific time period over which the weight loss occurred. This was treated as a separate time period for stratified analysis.

### 2.7 Primary Outcomes

The data from included studies were converted to two outcomes for meta‐analysis: The amount of weight lost (kg) and BMI change (primary outcomes). When weight loss was reported as a percentage, it was converted to kilograms. In several studies, baseline and diagnosis weights (or BMI values) were provided, enabling calculation of the change in weight or BMI.

### 2.8 Data Synthesis

Narrative and quantitative syntheses of evidence were conducted. Meta‐analyses of means were conducted separately for weight loss and BMI change to combine and evaluate the change prior to pancreatic cancer diagnosis. Pooled effect estimates with 95% confidence intervals were calculated for each outcome and were presented in separate forest plots.

### 2.9 Statistical Analysis

We assessed heterogeneity between studies using Cochran's Q‐statistic as well as Higgins and Thompson's inconsistency statistic (I^2^ index), with high or significant heterogeneity defined as *p* < 0.05 and I^2^ ≥ 75%, respectively [41, 42]. The I^2^ index provides the proportion of true variance in effect sizes over total observed variance. The null hypothesis of homogeneity of true effects was tested using the Q‐test. The significant heterogeneity between studies (likely due to the different study designs) resulted in using a random‐effects analysis to combine study results, as statistical homogeneity between studies could not be assumed [43, 44]. A fixed effects meta‐analysis was performed for each outcome as a sensitivity analysis to examine small‐study bias.

Sensitivity analysis (“Leave Out One”) was conducted to investigate the impact of each study on the summary estimate. Funnel plots [38, 45] and Egger's test [46] were evaluated to examine publication bias and possible small study effects (where smaller studies tend to show larger estimates of effect than larger studies). For this analysis, we used the ‘rma’ function in the ‘metafor’ package. Potential publication bias was indicated by asymmetry of the funnel plot and a *p*‐value of < 0.05 for Egger's test [46]. The contribution of each study to overall heterogeneity and its influence on the summary estimate were assessed through Baujat's plot [47].

Statistical analysis was conducted using RStudio version 2021.09.1 using the packages ‘meta’ (version 8.2–1), ‘metaviz’ (version 0.3.1) and ‘metafor’ (version 4.8–0) [48, 49].

### 2.10 Stratified Analysis

Stratified analysis, at the study level, was conducted for participant variables (moderators) which may influence the amount of weight lost: (1) diabetes status; (2) cancer type (PDAC or all types of pancreatic malignancies); (3) weight loss timing; (4) whether weight loss was self‐reported or obtained from medical records; and (5) percentage of late‐stage cases.

For stratification mixed‐effects meta‐analysis (random‐effects within subgroups, fixed‐effects between subgroups) with the Knapp and Hartung adjustment [50] was utilised due to the repeated analysis to reduce the likelihood of type I errors. The null hypothesis, that subgroups do not differ (i.e., that all moderators are unrelated to the effect sizes), was tested using a Q‐test based on analysis of variance (the omnibus test of model coefficients). The proportion of true variance explained by each factor (R^2^) was calculated to describe its influence on the weight loss and BMI change. In case of a significant Q‐test, post hoc pairwise comparisons were conducted. To allow for multiple comparisons, we used the Bonferroni correction [51] to reduce the danger of type I errors, by maintaining the overall type I error rate at *p* = 0.05 [52]. The significance level for weight loss (kg) was set at *p* < 0.01 to account for five stratified group comparisons. For BMI, the significance level was set at *p* < 0.0125, reflecting four comparisons due to insufficient data for stratified analysis of the percentage of late‐stage cases.

### 2.11 Subgroup Analysis

Subgroup analysis was conducted for possible confounding factors: (1) study design; (2) publication year; (3) country; (4) mean age; and (5) percentage of male participants.

## 3 Results

### 3.1 Study Characteristics

A total of 25 studies, comprising 25,971 participants, met the inclusion criteria and were included in the meta‐analysis. The main characteristics of the included studies are reported in Table 1. There were 20 studies that quantified weight loss and 10 studies reporting BMI change. All included studies were published after 2005. Further characteristics of the study participants for each included study are available in the Zenodo repository, https://doi.org/10.5281/zenodo.15555343.

**TABLE 1:** Characteristics of included studies.

Author and year	Title	DOI	Source	Country	Methodology	Study Design	Source of Data	Weight Loss Outcome	BMI Change Outcome
Babic et al. [57]	Adipose tissue and skeletal muscle wasting precede the clinical diagnosis of pancreatic cancer	10.1038/s41467‐023‐40, 024‐3	Nature Communications	USA	Restrospective analysis of records of people diagnosed with pancreatic cancer	Retrospective	Medical records	Included	Included
Bachmann et al. (2008)	Cachexia worsens Prognosis in Patients with Resectable Pancreatic Cancer	10.1007/s11605‐008‐0505‐z	Journal of Gastrointestinal Surgery	Germany	Prospective observational study of consecutive pancreatic cancer patients	Prospective Observational	Medical records	Included	Only BMI at diagnosis reported
Bibby et al. (2022)	From prehab to rehab: Nutritional support for people undergoing pancreatic cancer surgery	10.1111/jhn.13040	Journal of Human Nutrition and Dietetics	UK	A prospective study of patients with pancreatic and peri‐ampullary premalignant disease at the Manchester Royal Infirmary, UK	Prospective Observational	Patient recall or medical records when available	Included	Only BMI at diagnosis reported
Bye et al. (2013)	Symptoms in advanced pancreatic cancer are of importance for energy intake	10.1007/s00520‐012‐1514‐8	Supportive Care in Cancer	Norway	Patients with advanced unresectable pancreatic cancer were consecutively recruited	Prospective Observational	Recall and measurement at clinic follow‐ups	Included	Weight loss not BMI change reported
Chakedis et al. [56]	Identification of circulating plasma ceramides as a potential sexually dimorphic biomarker of pancreatic cancer‐induced cachexia	10.1002/rco2.68	JCSM Rapid Communications	USA	Patients asked about weight loss during pre‐operative visit	Prospective Observational	Patient recall verified by medical records where possible	Percentage weight loss reported without baseline	Included
Chari et al. (2008)	Pancreatic Cancer‐associated Diabetes Mellitus: Prevalence and Temporal Association with Diagnosis of Cancer	10.1053/j.gastro.2007.10.040	Gastroenterology	USA	Case–control review of medical records	Case–Control	Medical records	Included	Included
Dugnani et al. (2016a)	Diabetes associated with pancreatic ductal adenocarcinoma is just diabetes: Results of a prospective observational study in surgical patients	10.1016/jpan.2016.08.005	Pancreatology	Italy	Prospective observational study of PDAC patients admitted to a referral centre for pancreatic disease	Prospective Observational	Inpatient and outpatient medical records	Included	Weight loss not BMI change reported
Dugnani et al. (2016b)	Insulin resistance is associated with the aggressiveness of pancreatic ductal carcinoma	10.1007/s00592‐016‐0893‐6	Acta Diabetologica	Italy	Prospective observational study of patients admitted to a referral centre for pancreatic disease	Prospective Observational	Inpatient and outpatient medical records	Included	Weight loss not BMI change reported
Hart et al. (2011)	Weight Loss Precedes Cancer‐Specific Symptoms in Pancreatic Cancer‐Associated Diabetes Mellitus	10.1097/MPA.0b013e318220816a	Pancreas	USA	Review of medical records of all patients with PaCa living in Olmsted County and matched controls	Case–Control	Medical records	Included	Weight loss not BMI change reported
Hue et al. [23]	Weight Loss as an Untapped Early Detection Marker in Pancreatic and Periampullary Cancer	10.1245/s10434‐021‐09861‐8	Annals of Surgical Oncology	USA	Review of medical records with two matched controls	Case–Control	Medical records	Included	Weight loss not BMI change reported
Jabłońska et al. (2021)	Associations between Nutritional and Immune Status and Clinicopathologic Factors in Patients with Pancreatic Cancer: A Comprehensive Analysis	10.3390/cancers13205041	Cancers	Poland	Retrospective analysis of patients undergoing pancreatectomy	Retrospective	Asking patients	Included	Weight loss not BMI change reported
Jachnis and Slodkowski (2021)	The Relationship between Nutritional Status and Body Composition with Clinical Parameters, Tumour Stage, Ca19‐9, CEA Levels in Patients with Pancreatic and Periampullary Tumours	10.3390/curroncol28060406	Current Oncology	Poland	Prospective study of surgical patients undergoing treatment for pancreatic and periampullary tumours	Prospective Observational	Clinical Routine before surgery	No measure of dispersion reported	Included
Jeon et al. [53]	Prediction of Pancreatic Cancer in Diabetes Patients with Worsening Glycemic Control	10.1158/1055‐9965.EPI‐21‐0712	Cancer Epidemiology, Biomarkers and Prevention	USA	Review of records within VA health system	Prospective Observational (cohort)	Medical records	Included	Weight loss not BMI change reported
Lee et al. (2012)	Do new‐onset diabetes patients need pancreatic cancer Screening?	10.1097/MCG.0b013e318238348c	Journal of Clinical Gastroenterology	Korea	Review of medical records of cases of new‐onset diabetes (cases with PaCa and controls without PaCa)	Case–Control	Medical records	Included	Weight loss not BMI change reported
Lemanska et al. [25]	BMI and HbA1c are metabolic markers for pancreatic cancer: Matched case–control study using a UK primary care database	10.1371/journal.pone.0275369	Plos One	UK	Review of medical records of PaCa cases and matched controls	Case–Control	Medical records	BMI change not weight loss reported	Included
Ma et al. (2022)	Diabetes duration and weight loss are associated with onset age and remote metastasis of pancreatic cancer in patients with diabetes mellitus	10.1111/1753‐0407.13259	Journal of Diabetes	China	A retrospective study was conducted in patients with PC and DM hospitalised in Peking Union Medical College Hospital	Retrospective	Medical records	Included	Weight loss not BMI change reported
Nemer et al. (2017)	Predictors of Pancreatic Cancer‐Associated Weight Loss and Nutritional Interventions	10.1097/MPA.0000000000000898	Pancreas	USA	Retrospective review of medical records and evaluation of consecutive PDAC patients	Retrospective	Medical records and self‐reported when medical record information unavailable	Included	Included
Olson et al. (2016)	Weight Loss, Diabetes, Fatigue and Depression Preceding Pancreatic Cancer	10.1097/MPA.0000000000000590	Pancreas	USA	PDAC patients of Memorial Sloan Kettering Cancer Centre and controls	Case–Control	Interview	Included	Weight loss not BMI change reported
Pannala et al. [55]	Prevalence and Clinical Profile of Pancreatic Cancer‐Associated Diabetes Mellitus	10.1053/j.gastro.2008.01.039	Gastroenterology	USA	Patients with PaCa seen at Mayo Clinics and controls visiting Mayo Clinics for other reasons	Case–Control	Questionnaire and medical records	Included	Included
Ramsey et al. (2019)	Circulating interleukin‐6 is associated with disease progression, but not cachexia in pancreatic cancer	10.1016/j.pan.2018.11.002	Pancreatology	USA	Retrospective study of patients with biopsy proven PDAC	Retrospective	Usual body weight was self‐reported, other weights from clinical records	BMI change not weight loss reported	Included
Sah et al. [24]	Phases of Metabolic and Soft Tissue Changes in Months Preceding a Diagnosis of Pancreatic Ductal Adenocarcinoma	10.1053/j.gastro.2019.01.039	Gastroenterology	USA	Data from Rochester Epidemiology project	Case–Control	Medical records	Included	Weight loss not BMI change reported
Souza et al. (2007)	Analysis of Pancreatic Adenocarcinoma Tumour Staging and Resection according to Previous Body Mass Index and Diabetes Duration	10.1159/000104244	Pancreatology	Brazil	Consecutive patients admitted with PDAC	Retrospective	Medical records	BMI change not weight loss reported	Included
Talar‐Wojnarowska et al. (2020)	Clinical Significance of Activin A and Myostatin in Patients with Pancreatic Adenocarcinoma and Progressive Weight Loss	10.26402/jpp.2020.1.10	Journal of Physiology and Pharmacology	Poland	Prospective study of newly diagnosed PaCa cases with a healthy control group (not matched)	Prospective Observational (cohort)	Medical records and self‐reported	Included	Weight loss not BMI change reported
Trestini et al. (2020)	Prognostic Impact of Preoperative Nutritional Risk in Patients Who Undergo Surgery for Pancreatic Adenocarcinoma	10.1245/s10434‐020‐08515‐5	Annals of Surgical Oncology	Italy	Data from patients who underwent surgery for PaCa. Retrospective analysis of previously created prospective cohort	Retrospective	Medical records	Included	Included
Yuan et al. [54]	Diabetes, Weight Change and Pancreatic Cancer Risk	10.1001/jamaoncol.2020.2948	JAMA Oncology	USA	Cohort study of data from Nurses Health Study and Health Professionals Follow‐Up Study	Prospective Observational (cohort)	Surveys	Included	Weight loss not BMI change reported

*Note:* Reasons for the exclusion of studies for each outcome are given in the rightmost two columns.

Abbreviations: DM, Diabetes Mellitus; IQR, Interquartile Range; PaCa, Pancreatic Cancer; PDAC, Pancreatic Ductal Adenocarcinoma; SD, Standard Deviation; SE, Standard Error.

Although weight loss is widely known to be associated with pancreatic cancer, we found that there were few studies that quantify pre‐diagnostic weight loss in pancreatic cancer. This resulted in a relatively low number of studies eligible for inclusion. To this effect, from 328 articles which remained after the initial screening, only 25 studies were included in the final analysis. The main reasons for exclusion at the final screening stage included grouping all gastrointestinal cancers together, reporting unquantified weight loss (i.e., present or absent), not reporting measures of dispersion such as standard deviation, only presenting weight loss graphically, and only reporting the results of regression analysis. Many studies also focused on people with new‐onset diabetes and reported weight or BMI at diabetes onset instead of prior to pancreatic cancer diagnosis.

### 3.2 Weight Loss

**FIGURE 2:**
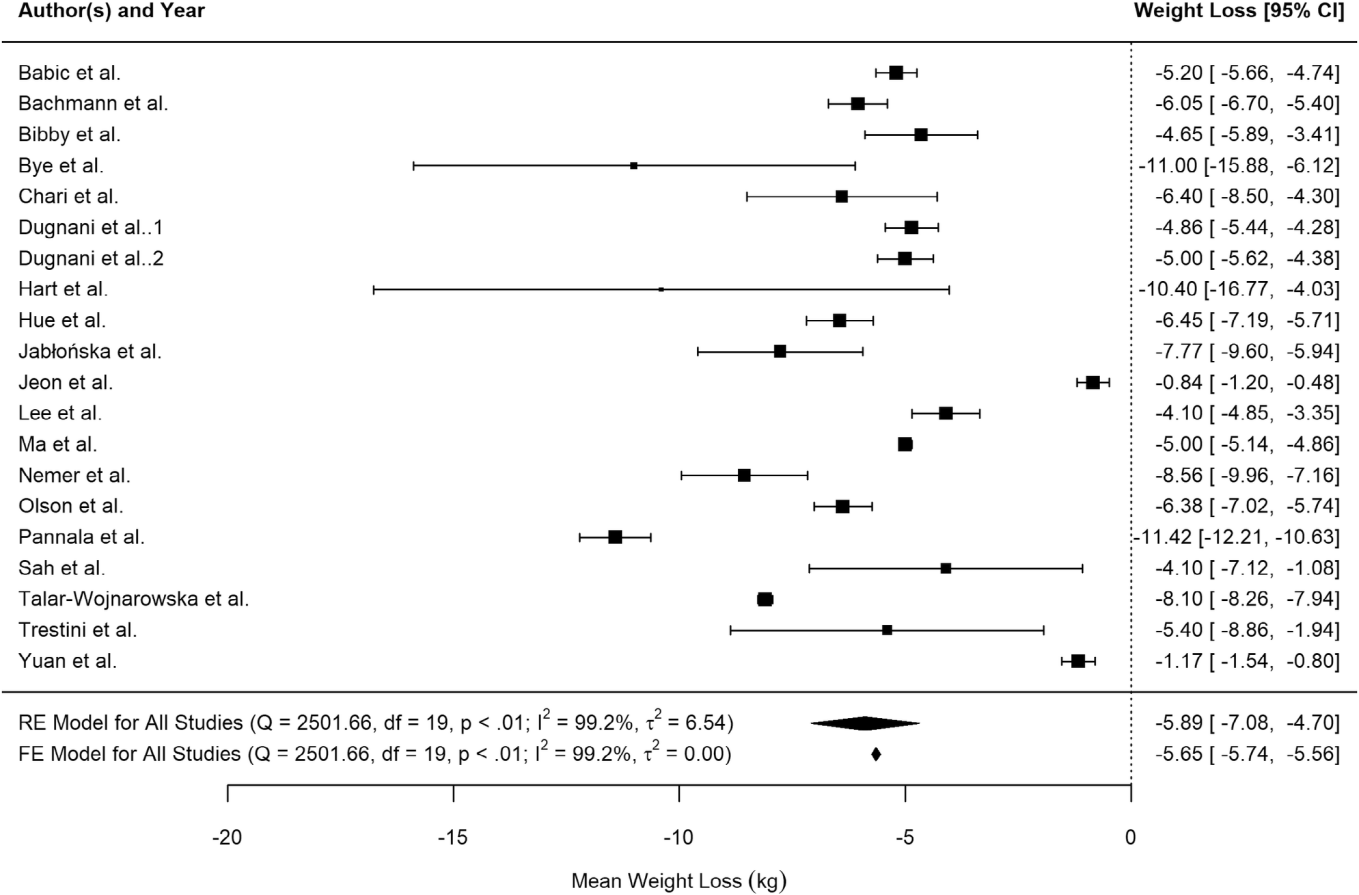
Forest plot of random‐effects meta‐analysis of weight loss in kg prior to pancreatic cancer diagnosis.

Twenty studies reported weight loss before pancreatic cancer diagnosis, or information which allowed weight loss to be calculated (e.g., baseline weight and diagnosis weight). Figure 2 shows the results of the random‐effects meta‐analysis quantifying weight loss in kg prior to pancreatic cancer diagnosis at 5.9 kg [95% CI 4.7 to 7.1]. There was considerable and significant heterogeneity between studies (I^2^ = 99.2% (tau‐based), *p* < 0.001). Neither Baujat's plot nor Leave Out One sensitivity analysis showed that any one study unduly influenced heterogeneity nor the summary estimate.

The studies by Jeon et al. [53], and Yuan et al. [54], which reported the smallest weight loss, were also the largest and second‐largest studies, raising potential concerns about small study effects. However, funnel plot analysis and Egger's test (*p* = 0.086) did not support this concern. In contrast, the third‐largest study by Pannala et al. [55], reported the highest weight loss, and fixed‐effects meta‐analysis produced a similar central estimate, with a narrower prediction interval as expected. Overall, the random‐effects meta‐analysis was unlikely to be influenced by small study bias.

### 3.3 BMI Change

**FIGURE 3:**
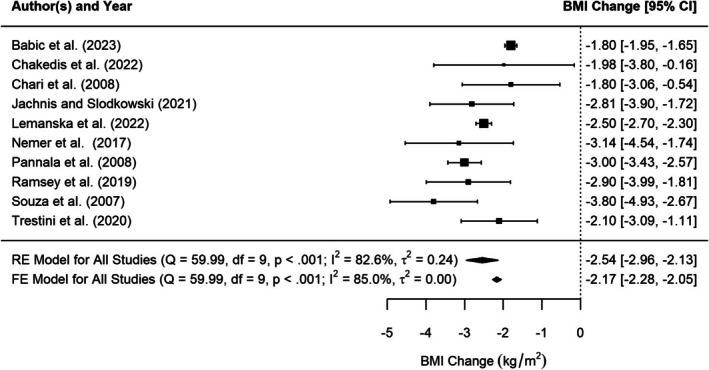
Forest plot of random‐effects meta‐analysis of BMI change prior to pancreatic cancer diagnosis.

Ten studies reported BMI change, or information which allowed this to be calculated (e.g., baseline BMI and diagnosis BMI), prior to pancreatic cancer diagnosis. Figure 3 shows the results of the random‐effects meta‐analysis of BMI change prior to pancreatic cancer diagnosis with a change of −2.5 kg/m^2^ [95% CI −3.0 to −2.1]. There is significant heterogeneity (I^2^ = 82.6% (tau‐based), *p* < 0.001). Neither Baujat's plot nor Leave Out One sensitivity analysis showed that any one study unduly influenced heterogeneity nor the summary estimate.

The study by Chakedis et al. [56], had a wide confidence interval, which was expected given its small sample size (*n* = 53) and resulting low precision. In contrast, the studies by Lemanska et al. [25], and Babic et al. [57], the two largest, showed narrow confidence intervals (as expected from larger studies). Funnel plot analysis and Egger's test (*p* = 0.454) provided no evidence of publication bias or small study effects. Similarly, the fixed‐effects meta‐analysis yielded a comparable central estimate with a narrower prediction interval, as expected. Thus, the random‐effects meta‐analysis result was unlikely to be influenced by small study bias.

### 3.4 Stratified Analysis

For both outcomes, the omnibus Q‐test shows that at least part of the heterogeneity between the studies in the true effects was related to the moderators of diabetes status, pancreatic cancer type, weight loss timing and whether weight loss was self‐reported (*p* < 0.001). The percentage of late‐stage cases did not account for any heterogeneity for weight loss and could not be analysed for BMI change, as it was reported by too few studies.

#### 3.4.1 Diabetes Status

Diabetes status accounted for 0.15% of the heterogeneity for weight loss. There were three stratification levels: Studies where all participants had diabetes (5 studies), studies where some participants had diabetes (11 studies) and studies where the diabetes status of the participants was unknown (4 studies). Post hoc pairwise comparisons revealed no significant difference (*p* = 0.264 to 0.952) between the effects pooled within studies where all participants had diabetes −4.6 kg [95% CI −7.3 to −1.9], where some participants had diabetes −6.3 kg [95% CI −8.0 to −4.6] and where the diabetes status of participants was unknown −6.2 kg [95% CI −9.2 to −3.2]. Although studies where all participants had diabetes had a lower estimate of effect, all of the estimates of effect were broadly similar. Therefore, it is not possible to determine whether individuals with diabetes lose more or less weight than those without diabetes, only that diabetes status influences the amount of weight lost.

Diabetes status did not account for any heterogeneity in BMI change. There were three stratification levels: Studies where all participants had diabetes (1 study), studies where some participants had diabetes (7 studies), and studies where the diabetes status of the participants was unknown (2 studies). Post hoc pairwise comparisons revealed no significant difference (*p* = 0.345 to 0.784) between the effects pooled within studies where all participants had diabetes −1.8 kg/m^2^ [95% CI −3.7 to 0.1], where some participants had diabetes −2.6 kg/m^2^ [95% CI −3.2 to −2.1], and where diabetes status of participants was unknown −2.5 kg/m^2^ [95% CI −3.7 to −1.3]. As fewer studies contributed data to the subgroups where all participants had diabetes or where diabetes status was unknown (compared with the subgroup with mixed or known diabetes status), this analysis is unlikely to have yielded meaningful findings.

#### 3.4.2 Pancreatic Cancer Type

For weight loss, whether the pancreatic cancer type in the study was PDAC (12 studies) or all pancreatic malignancies (8 studies) did not account for any heterogeneity. The effect estimates were similar between studies with participants who only had PDAC: −5.8 kg [95% CI −7.5 to −4.1] and those where participants had all types of pancreatic malignancies: −6.0 kg [95% CI −8.1 to −4.0]. Post hoc comparison showed no significant difference between these groups (*p* = 0.862).

For BMI change, whether pancreatic cancer type was PDAC (5 studies) or all pancreatic malignancies (5 studies), accounted for 1.3% of heterogeneity. As found for weight loss effect estimates, they were similar between studies with participants who only had PDAC −2.8 kg/m^2^ [95% CI −3.6 to −2.1] and those where participants had all types of pancreatic malignancies −2.4 kg/m^2^ [95% CI −3.0 to −1.8]. Post hoc comparison showed no significant difference between these groups (*p* = 0.325).

#### 3.4.3 Weight Loss Timing

**FIGURE 4:**
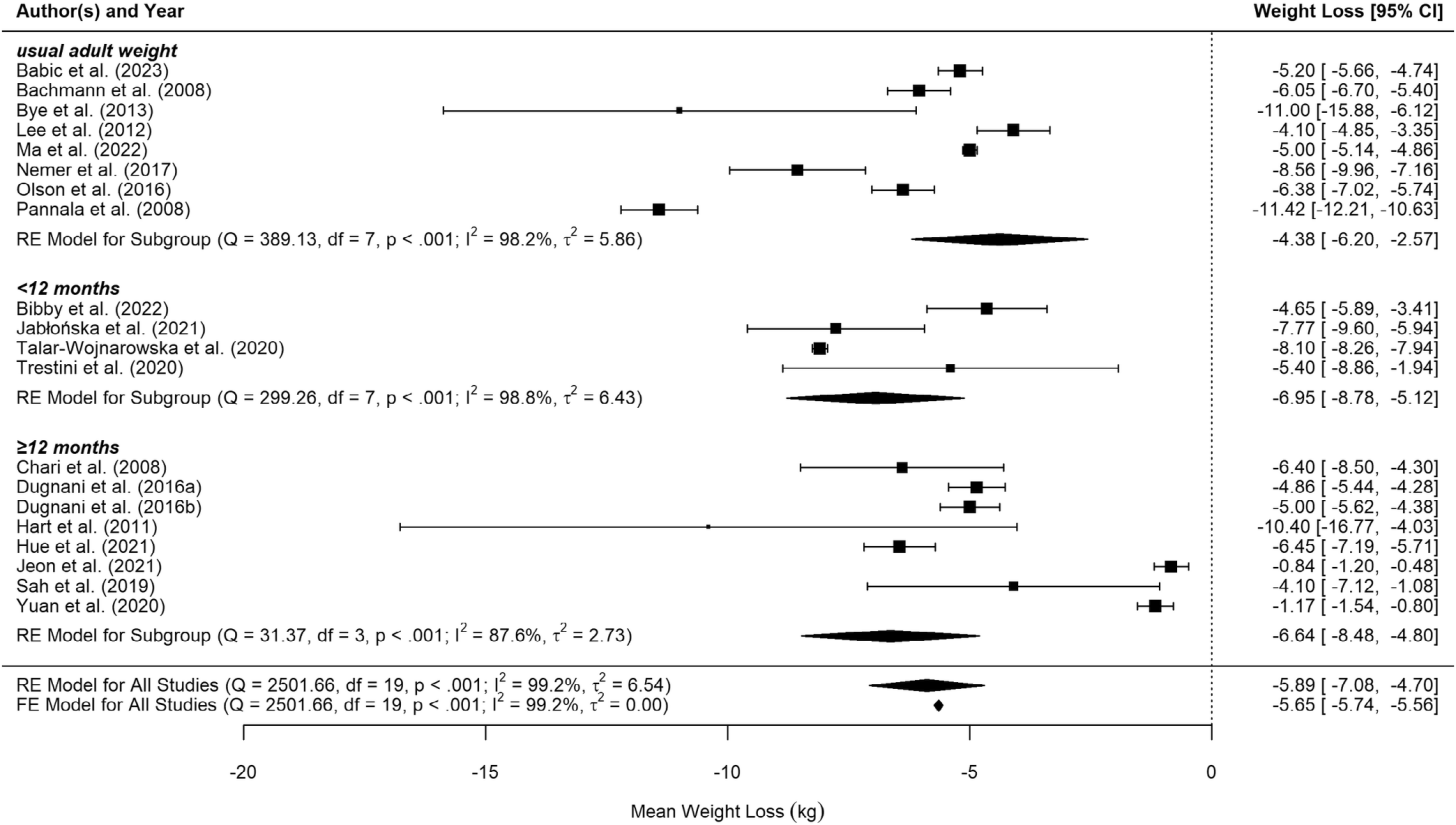
Forest plot of random‐effects meta‐analysis of weight loss in kg prior to pancreatic cancer diagnosis, stratified by weight loss timing.

Weight loss timing accounted for 15.5% of the heterogeneity for weight loss. There were three stratification levels for weight loss: Usual adult weight (8 studies), weight loss < 12 months before diagnosis (5 studies), and weight loss ≥ 12 months before diagnosis (7 studies), Figure 4. Post hoc pairwise comparisons revealed no significant difference (*p* = 0.061 to 0.827) between weight loss reported < 12 months before diagnosis −6.6 kg [95% CI −9.3 to −3.9], weight loss reported ≥ 12 months before diagnosis −4.4 kg [95% CI −6.3 to −2.4], and weight loss reported from usual adult weight −6.9 kg [95% CI −8.8 to −5.1]. The effect estimate is higher for weight loss closer to diagnosis.

To investigate further, we repeated the stratified analysis with six stratification levels: Usual adult weight (8 studies), weight 48 months before diagnosis (1 study), weight 12 months before diagnosis (7 studies), weight nine months before diagnosis (1 study), weight six months before diagnosis (2 studies) and weight 4 months before diagnosis (1 study). Post hoc comparisons only showed a significant difference between weights reported at 48 months −6.4 kg [95% CI −12.3 to −0.5] and usual weight reports −6.9 kg [95% CI −8.9 to −4.9] (*p* = 0.001). The other groups did not significantly differ from one another (*p* = 0.061 to 0.968). The largest effect estimate was seen for weights reported 4 months before diagnosis −7.8 kg [95% CI −13.6 to −2.0], and the smallest for weights reported 12 months before diagnosis −4.1 kg [95% CI −6.1 to −2.1]. This again may indicate that people lose more weight closer to diagnosis, which would be expected. However, due to the highly fragmented stratification, with many levels containing only 1 or 2 studies, these findings are not robust.

Weight loss timing accounted for none of the heterogeneity for BMI change. There were three stratification levels for BMI change: Usual adult weight (5 studies), weight loss < 12 months before diagnosis (2 studies) and weight loss ≥ 12 months before diagnosis (3 studies). Post hoc comparison showed no significant differences between the groups (*p* = 0.514 to 0.910). We then repeated the analysis with five stratification levels for BMI change from: Usual adult BMI (5 studies), BMI 48 months before diagnosis (1 study), BMI 12 months before diagnosis (2 studies), BMI 6 months before diagnosis (1 study) and BMI 3.5 months before diagnosis (1 study). The largest BMI change was for BMI reported 3.5 months before diagnosis −3.8 kg/m^2^ [95% CI −5.5 to −2.1] and at least 48 months before −1.8 kg/m^2^ [95% CI −3.6 to 0.0]. As with the weight loss outcome, the effect estimate is highest for weight loss nearer diagnosis, and this may again indicate that people lose more weight closer to diagnosis, which would be expected.

#### 3.4.4 Self‐Reported Weight Change

**FIGURE 5:**
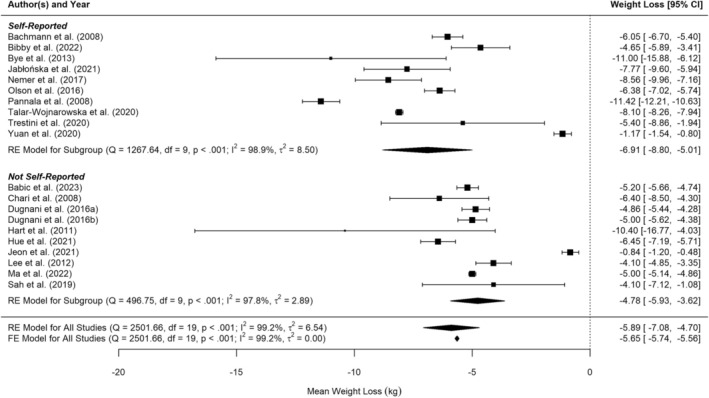
Forest plot of random‐effects meta‐analysis of weight loss in kg prior to pancreatic cancer diagnosis, stratified by whether weight loss was self‐reported.

Whether weight loss was self‐reported (10 studies) or obtained from medical records (10 studies) accounted for 11.4% of the heterogeneity for the amount of weight loss, Figure 5. Post hoc comparison between self‐reported weight loss −6.9 kg [95% CI −8.5 to −5.2] and weight loss obtained from medical records −4.8 kg [95% CI −6.5 to −3.3] was not significant (*p* = 0.100). However, this may still suggest that self‐reported weight loss tends to be greater than weight loss calculated from medical records, indicating potential recall bias.

**FIGURE 6:**
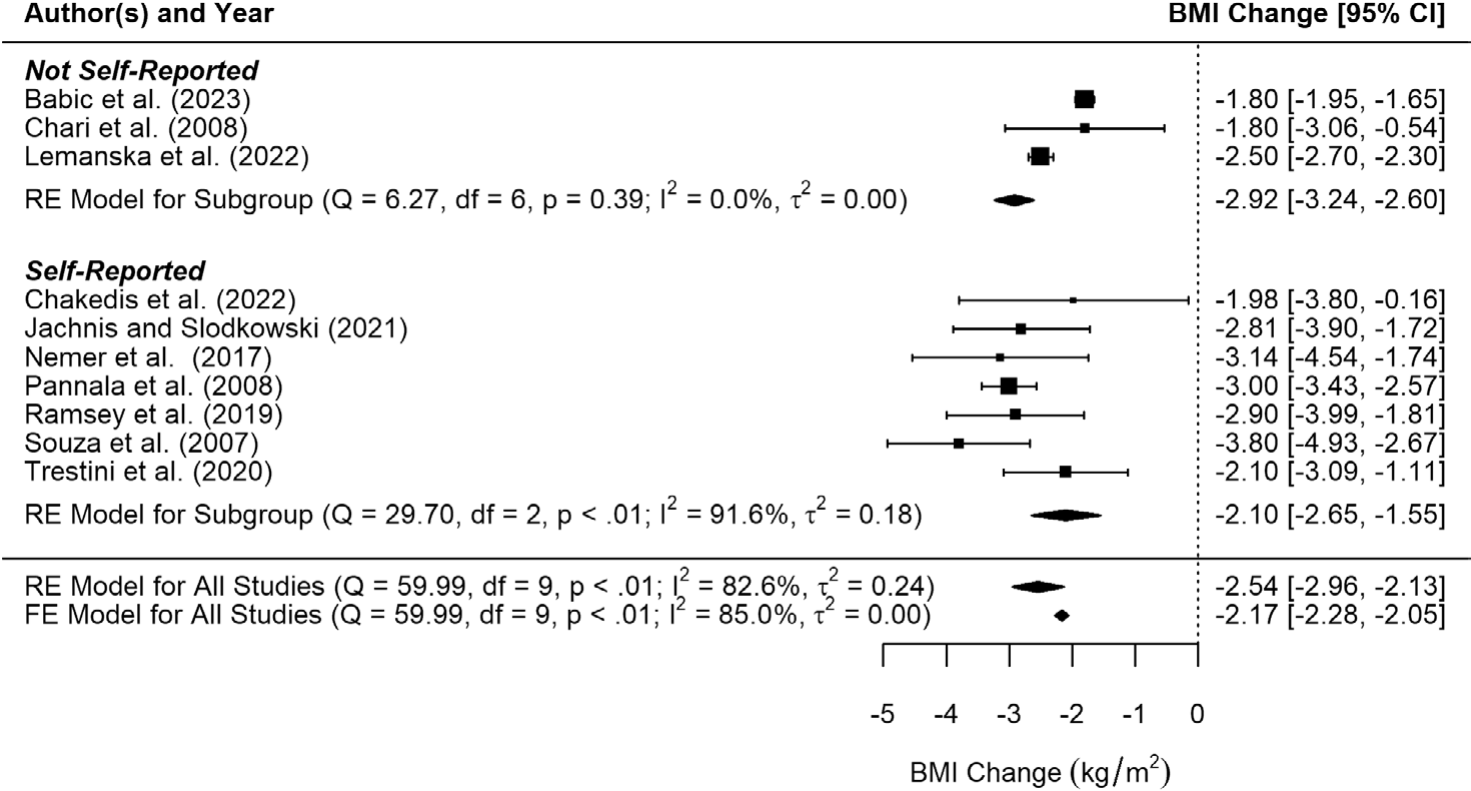
Forest plot of random‐effects meta‐analysis of BMI change prior to pancreatic cancer diagnosis, stratified by whether BMI change was self‐reported.

Whether the BMI change was self‐reported (7 studies) or obtained from medical records (3 studies) accounted for 49.6% of the heterogeneity, Figure 6. The post hoc comparison between the pooled effect estimates of self‐reported BMI change −2.9 kg/m^2^ [95% CI −3.4 to −2.4] and BMI change from medical records −2.1 kg/m^2^ [95% CI −2.6 to −1.6] was not significant (*p* = 0.035), using the Bonferroni correction. This may still indicate that self‐reported BMI change is greater than BMI change calculated from medical records.

### 3.5 Subgroup Analysis

For both weight loss and BMI change, the omnibus Q‐test shows that at least part of the heterogeneity in the true effects was related to the possible confounding factor of publication year (*p* < 0.001). Publication year accounted for 13.1% of the heterogeneity for weight loss and for 49.5% for BMI. For both outcomes, more recent studies report less weight loss compared with older studies. For weight loss, study design accounted for part of the heterogeneity (6.6%) but not for BMI change (*p* < 0.001).

The percentage of male participants in a study accounted for 25.8% of the heterogeneity for weight loss (*p* < 0.001), with studies with lower percentages of male participants reporting greater amounts of weight loss. The percentage of male participants did not account for any heterogeneity for BMI. Neither the mean age of study participants nor the country in which the study was conducted accounted for any heterogeneity for either outcome.

## 4 Discussion

### 4.1 Summary

In this systematic review and meta‐analysis of 25 studies (*n* = 25,971), mean weight loss before diagnosis was 5.9 kg and mean BMI change was −2.5 kg/m^2^. Substantial between‐study heterogeneity was observed. This was partially explained by self‐reported weights vs. those obtained from medical records and publication year for both outcomes; additionally, diabetes status and timing of weight loss contributed to heterogeneity for the weight change outcome, while pancreatic cancer type contributed to heterogeneity in the BMI change outcome. Overall, these findings support that weight loss commonly precedes pancreatic cancer diagnosis.

### 4.2 Findings in the Context of Previous Research

Although pancreatic cancer progresses rapidly, weight loss has potential value as an early diagnostic marker and is increasingly incorporated into predictive models and algorithms [29, 30, 31]. Previous work by Lemanska et al. [25], found that weight loss is significant up to two years before pancreatic cancer diagnosis, with Sah et al. [24], finding weight and soft tissue changes occurring up to 5 years before diagnosis. This indicates there is a clinically meaningful window for earlier detection. Earlier diagnosis is critical as detection even six months sooner [58] may increase the likelihood of diagnosis of a resectable and therefore curable disease.

The studies included here assessed weight change across heterogeneous timeframes (3.5 to 48 months or “usual weight”), precluding identification of an optimal diagnostic interval. More frequent and standardised recording of weight or BMI would improve quantification of weight loss before pancreatic cancer diagnosis and strengthen trend‐based detection approaches [58]. However, this analysis also highlights limitations in routine medical data, where weight and BMI are often only opportunistically recorded.

Unintentional weight loss is already incorporated into referral criteria for suspected pancreatic cancer in individuals with new‐onset diabetes in the NHS [31]. Our findings indicate that pre‐diagnostic weight loss varies by diabetes status, although there is insufficient evidence about the direction of this effect. Previous work suggests greater weight loss in individuals with both diabetes and pancreatic cancer compared with pancreatic cancer alone [55]. These findings reinforce the importance of new‐onset diabetes accompanied by weight loss as a key indication for referral for further investigation.

### 4.3 Clinical Implications

This meta‐analysis quantifies pre‐diagnostic weight loss and supports its role as a potential tool for earlier detection. Currently, one in three patients requires three or more GP visits before referral [17, 59], reflecting the lack of specific early markers. Weight loss is a useful clinical signal because it can precede hallmark symptoms such as jaundice, is frequently recorded, and is simple, inexpensive, and non‐invasive to monitor [60, 61].

However, weight loss is non‐specific and often multifactorial. It should therefore be considered within more comprehensive early diagnosis strategies rather than as a standalone marker. Weight changes are already used as part of the diagnosis strategy [31] and are already incorporated into several risk algorithms [29, 30, 32, 62]. Improved quantification could enhance their predictive accuracy. Such approaches may also help address heterogeneity identified in this study, including variation by diabetes status, pancreatic cancer type, weight loss timing, whether weight loss is self‐reported or obtained from medical records, and the percentage of male participants (sex distribution).

### 4.4 Strengths and Limitations

To our knowledge, this is the first meta‐analysis quantifying weight loss prior to pancreatic cancer diagnosis. By synthesising both absolute weight change and BMI across diverse study designs, we provide a comprehensive estimate of pre‐diagnostic weight loss.

Heterogeneity was substantial and likely reflects variation in study design, populations, and outcome definitions. Included studies ranged from large population‐based cohorts to smaller samples from individual healthcare providers, with differing baseline health status and healthcare engagement. Variation in measurement approaches, including self‐reported weights, use of “usual adult weight,” and inconsistent timepoints, further contributed to heterogeneity and potential recall bias.

Weight loss trajectories are unlikely to be linear, with a more rapid decline closer to diagnosis. We therefore did not standardise weight loss per unit time to avoid distorting temporal patterns. Additionally, variation in the timing of diagnosis introduces bias, as weight change from disease onset cannot be directly observed. Although the proportion of late‐stage cases might be expected to correlate with greater weight loss, stage did not explain heterogeneity in this analysis. Nonetheless, cancer stage at diagnosis remains a crucial factor to record and consider when using weight loss as a diagnostic marker.

Differences between weight loss and BMI change outcomes likely reflect measurement characteristics. BMI is an indirect measure and a relatively crude indicator often recorded in clinical settings, while weight provides a direct estimate and is more often self‐reported. These differences may also explain why stratification variables accounted for different proportions of heterogeneity across outcomes.

Overall, stratification highlighted sources of heterogeneity but did not adequately explain between‐study heterogeneity. Subgroup analysis for possible confounding factors indicated that publication year partly explained some heterogeneity, though moderate heterogeneity persisted (I^2^ > 50%). These analyses were conducted at the study level using aggregate data and therefore do not permit direct inference about differences between individuals due to diabetes status or sex.

As this meta‐analysis is based on observational studies, the certainty of evidence is inherently limited under GRADE (Grading of Recommendations, Assessment, Development, and Evaluations) criteria [63, 64]. However, the population‐based studies enhance generalisability, as they did not include highly selected populations. Conversely, residual unexplained heterogeneity remained, and only 10% of full‐text articles were screened in duplicate, which may have introduced selection bias and limited the reliability of the study selection process. Therefore, the certainty of evidence is moderate.

### 4.5 Future Research

Further research is needed to refine estimates of pre‐diagnostic weight loss. Existing studies are limited in number and highly heterogeneous. Standardised reporting, preferably using BMI, would improve comparability between studies [65, 66]. Frequent measurements (e.g., every three months in the year preceding diagnosis) derived from medical records are recommended to better define the onset and trajectory of weight loss and minimise recall bias [67].

Future studies should report measures of dispersion and key modifiers, including diabetes status (type and duration), pancreatic cancer subtype, weight loss timing and stage at diagnosis. Stratified analyses by sex and diabetes status are also needed to help to clarify whether the magnitude of weight loss is consistent across key clinical subgroups. This will improve the clinical applicability of the findings and enhance the utility of weight loss as part of early detection strategies.

## 5 Conclusions

Despite the association between weight loss and pancreatic cancer being widely recognised, to our knowledge, this systematic review is the first that provides a comprehensive meta‐analysis to quantify weight loss prior to pancreatic cancer diagnosis. We found considerable heterogeneity in reporting between studies. We concluded that to strengthen the quality of evidence, international standards of reporting of weight and weight loss need to be improved.

## Author Contributions

**Nadia A. S. Smith:** conceptualization, methodology, formal analysis, supervision, writing – review and editing. **Claire A. Price:** conceptualization, methodology, data curation, formal analysis, visualization, project administration, writing – original draft, writing – review and editing. **Freda Mold:** methodology, writing – review and editing, supervision. **Agnieszka Lemanska:** conceptualization, methodology, formal analysis, funding acquisition, project administration, writing – review and editing, supervision. **Debbie Cooke:** conceptualization, supervision. **Martyn Winn:** methodology, writing – review and editing, supervision.

## Funding

This project is funded as part of an EPSRC iCase studentship awarded to CP (Project Reference: 2702921). The work of NPL co‐authors was funded by the UK Government's Department for Science, Innovation & Technology through the UK's National Measurement System programmes. For the purpose of Open Access, the author has applied a Creative Commons Attribution (CC BY) public copyright licence to any Author Accepted Manuscript version arising from this submission.

## Ethics Statement

The authors have nothing to report.

## Conflicts of Interest

The authors declare no conflicts of interest.

## Supporting information


**Supporting Information: 1** Deviations from Protocol.docx.
Explanation of minor deviations from the PROSPERO protocol.

**Supporting Information: 2** PRSIMA Checklist.docx.

**Supporting Information: 3** PRISMA Abstract Checklist.docx.

**Supporting Information: 4** Search Strategy.docx.

**Supporting Information: 5** ROBINS‐I Quality Assessment.xlsx.
Spreadsheet of the quality assessment of eligible studies using ROBINS‐I.

## Data Availability

The data that support the findings of this study are openly available in Zenodo at https://doi.org/10.5281/zenodo.15555343.
