# Supplementary material for: Quantifying Weight Loss Prior to Pancreatic Cancer Diagnosis: A Systematic Review and Meta‐Analysis
Source: Cancer Med. 2026 May 31;15(6):e71997. doi: 10.1002/cam4.71997 (PMC13239862; doi:10.1002/cam4.71997)
Supplement: Supplementary file 1 — Supporting Information: 1 Deviations from Protocol.docx. Explanation of minor deviations from the PROSPERO protocol. [file CAM4-15-e71997-s001.docx]

S1 Deviations from Protocol

In conducting this review there have been minor deviations from the PROSPERO protocol. It was intended to analyse odds ratios (OR) and hazard ratios (HR), however only 6 studies reported either OR or HR, so there was insufficient data to perform this meta-analysis. It was also intended that we analyse each study type separately. However, due to the number of different outcomes found, analysis was conducted with all studies for an outcome with subgroup analysis conducted to assess heterogeneity due to study type.

Stratified analysis was also planned for diabetes onset time, but this was only reported by three studies. Similarly, stratified analysis was planned for cachexia (weight loss seen in many people with cancer later in the disease progression) status, but this was only reported by four studies. Therefore, there were not enough studies reporting these to enable these stratification analyses to be undertaken.
