# Supplementary material for: Quantifying Weight Loss Prior to Pancreatic Cancer Diagnosis: A Systematic Review and Meta‐Analysis
Source: Cancer Med. 2026 May 31;15(6):e71997. doi: 10.1002/cam4.71997 (PMC13239862; doi:10.1002/cam4.71997)
Supplement: Supplementary file 4 — Supporting Information: 4 Search Strategy.docx. [file CAM4-15-e71997-s003.docx]

S4 Search Strategy

**Interface – EBSCOhost Research Database**

**Search Screen – Advanced Search**

**Database – Medline**

| **1** | (Pancreas OR Pancreatic) ab.ti |
| --- | --- |
| **2** | (Ductal Adenocarcinoma OR PDAC OR Cancer OR Malignancy OR Adenocarcinoma OR Tumour OR Tumor) ab.ti |
| **3.** | (Weight OR BMI OR Body Mass Index) ab.ti |
| **4.** | (loss OR decrease OR change) ab.ti |
| **5.** | diagnos* ab.ti |

This search strategy was used for all databases. The equivalent abstract/title field was selected for all databases. For Web of Science this was the topic field, for Embase this was the title/abstract/author keywords field, for Scopus this was the title/abstract/keywords field, and for Cochrane this was the title/abstract/keyword field.
